# The role of the ubiquitin-proteasome pathway in rhTRIM5α restriction of HIV-1

**DOI:** 10.1186/1758-2652-13-S3-O9

**Published:** 2010-11-04

**Authors:** CM Danielson, TJ Hope

**Affiliations:** 1 Department of Cell and Molecular Biology, Northwestern University, Chicago, IL, 60611, USA

## Background

rhTRIM5α is a restriction factor that blocks HIV-1 infection by interacting with the capsid core early after entry. Although rhTRIM5α blocks accumulation of reverse transcription products, proteasome inhibitors rescue reverse transcription while maintaining a block to infection, supporting the involvement of the proteasome in the mechanism of restriction.

## Methods

First, rhTRIM5α was examined by immunofluorescence and characterized with specific ubiquitin antibodies. Next, mutations were made in rhTRIM5α to prevent involvement in the ubiquitin-proteasome pathway, and mutants were analyzed by immunofluorescence, flow cytometry and real-time PCR. A mutant virus was constructed to prevent ubiquitination of capsid, and used to infect cells expressing rhTRIM5α to characterize restriction and reverse transcription. Finally, a fluorescently tagged subunit of the proteasome, LMP2-GFP, was co-expressed with rhTRIM5α and analyzed by fixed and live cell microscopy.

## Results

Staining with specific ubiquitin antibodies revealed that rhTRIM5α cytoplasmic bodies contained polyubiquitinated proteins, but proteasome inhibitors decreased polyubiquitinated proteins within cytoplasmic bodies. rhTRIM5α mutants showed reduced restriction of HIV-1, and localized to cytoplasmic bodies with different patterns of ubiquitination. The mutant virus exhibited reduced infectivity and an intermediate phenotype with regard to the block of reverse transcription. Analysis of fluorescently tagged proteasomes in cells expressing rhTRIM5α showed that proteasomes were recruited to rhTRIM5α cytoplasmic bodies in the presence of virus, and live cell microscopy revealed interactions between virus and proteasomes.

## Conclusions

The relocalization of polyubiquitinated proteins upon proteasome inhibition may indicate an efflux of polyubiquitinated proteins, implicating cytoplasmic bodies in the process of ubiquitination. Analysis of rhTRIM5α mutants revealed that rhTRIM5α may play a partial role in conjugating and receiving ubiquitination. The mutant virus showed that ubiquitination of capsid is not required for rhTRIM5α restriction, but reverse transcription is affected. Finally, proteasomes are recruited to rhTRIM5α cytoplasmic bodies in the presence of virus, which may lead to proteasomal destruction of rhTRIM5α-virus complexes.

